# Linking the effect of psoriatic arthritis-related foot involvement to the Leeds Foot Impact Scale using the International Classification for Functioning, Disability and Health: a study to assess content validity

**DOI:** 10.1186/s13047-020-00420-0

**Published:** 2020-08-24

**Authors:** Kate Carter, Caterina Tannous, Steven Walmsley, Keith Rome, Deborah E. Turner

**Affiliations:** 1 Podiatry department, School of Health Science, Campbelltown Campus, Western Sydney University, Sydney, Australia; 2 Occupational therapy department. School of Health Science, Campbelltown Campus, Western Sydney University, Sydney, Australia; 3 Health and Rehabilitation Research Institute, Faculty of Health and Environmental Science, AUT University, 90 Akoranga Drive, Northcote, Auckland, 0627 New Zealand; 4 Podiatry department, School of Clinical Sciences, Kelvin Grove Campus, Queensland University of Technology, Brisbane, Queensland Australia

**Keywords:** Psoriatic arthritis, Foot, Patient reported outcome measures, International classification for functioning, Disability and health

## Abstract

**Background:**

Previous research to describe the impact of foot involvement in psoriatic arthritis has used the Leeds Foot Impact Scale in Rheumatoid Arthritis (LFIS-RA) in the current absence of any psoriatic arthritis foot-specific tools. However, the LFIS-RA is a rheumatoid arthritis disease-specific outcome measure and its content validity for evaluating the experiences of people with psoriatic arthritis-related foot involvement is unknown. The study objective was to determine the content validity of the LFIS-RA for assessing people with psoriatic arthritis, using the International Classification of Functioning, Disability and Health (ICF) as the frame of reference.

**Method:**

Concepts within each item of the LFIS-RA were linked to the best-matched ICF categories using established linking rules, which enable a systematic and standardised linking process. All concepts were independently linked to the ICF by 2 investigators with different professional backgrounds, which included occupational therapy and podiatry. The list of ICF categories derived from previous research that pertained to the foot in psoriatic arthritis was then compared with the ICF categories linked to the LFIS-RA. The comparison was undertaken in order to determine the extent to which concepts important and relevant to people with psoriatic arthritis-related foot involvement were addressed.

**Results:**

Thirty-five distinct ICF categories were linked to the LFIS-RA, which related to body functions (44%), activities and participation (35%), environmental factors (16%) and body structure (5%). In comparison with the ICF categories derived from concepts of the foot in psoriatic arthritis previously defined, the LFIS-RA provided coverage of key constructs including pain, functioning, daily activities, footwear restrictions and psychological impact. Other concepts of importance in psoriatic arthritis such as skin and toenail involvement, self-management and paid employment were not addressed in the LFIS-RA.

**Conclusion:**

Content validity of the LFIS-RA to determine the impact of foot functional impairments and disability in people with psoriatic arthritis was not supported by the results of this study. Future work should consider the development of a psoriatic arthritis foot-specific patient reported outcome measure, using the LFIS-RA as an important foundation.

## Background

Treating inflammatory arthritis as early as possible to minimise structural joint damage and functional disability has been shown to be effective in psoriatic arthritis (PsA) [1–3]. This approach requires precise evaluation of disease activity, functioning and response to therapy through validated outcome measures that incorporate the patient perspective to capture the full disease burden [4]. Historically, instruments developed to assess rheumatoid arthritis have been used in this patient group in the knowledge of limitations which include disparities in; pathophysiology, patterns of joint involvement, cutaneous manifestations, range of musculoskeletal features [5–7], as well as differences in the impact of the diseases on health-related quality of life [8, 9].

Disease persistence in the foot with potential to progress to structural joint damage has been found in the context of low global disease activity in PsA [10], which indicates the need for foot-specific outcome tools. In the absence of a validated disease and foot region-specific outcome measure to assess the impact of localised disease in the foot in PsA, the Leeds Foot Impact Scale in rheumatoid arthritis (LFIS-RA) has been used in previous studies [10–15]. However, how well the LFIS-RA functions and measures what is intended to be measured in PsA is not known.

The LFIS-RA is a validated patient-reported outcome measure developed specifically to assess foot-related impairment and disability in rheumatoid arthritis [16], with content generated from semi-structured interviews among 30 people with rheumatoid arthritis and content validity assessed by subsequent postal surveys. The constructs assessed by the LFIS-RA are closely associated with the components of the International Classification of Function, Health and Disability (ICF), providing a strong conceptual basis [17]. The LFIS-RA has demonstrable measurement properties and it has been suggested as a valuable measure, alongside other core outcomes, to help determine objective treatment targets for tight control of foot-specific disease activity in rheumatoid arthritis [18, 19]. The extent to which a patient-reported outcome measure adequately assesses constructs relating to disease conditions or associated phenomena is known as content validity. The content validity of an outcome measure has been asserted by an international working group in outcome measurement instruments as the most important of all the required measurement properties [20]. However, the level of content validity of the LFIS-RA for use in PsA and how well it reflects the impact of foot involvement on functioning and participation typical for people with PsA is currently unknown.

The ICF provides a common framework that can be used to evaluate the conceptual coverage of items and aspects of content validity for outcome measures used in specific diseases [21, 22]. Furthermore, the Outcome Measures in Rheumatology (OMERACT) groups have used the ICF as a universal framework to define ‘what to measure’ when assessing the impact of disease on functioning [23], and the content of items in outcome measures have been linked to the ICF classification to validate the ‘truth’ component of the OMERACT Filter [21, 22, 24]. The ‘truth’ section of the OMERACT Filter requires that the outcome instrument meets the criteria for content, face and construct validity [24]. It has been suggested that researchers and clinicians looking for instruments should first identify an outcome according to the concepts relevant to people with PsA and then select an instrument that covers the identified outcome [25]. Concepts important and relevant to people with foot problems in PsA have recently been identified and linked to the ICF classification to comprehensively define what should be measured in the evaluation of PsA-specific foot disease burden [26]. This presented the opportunity to gain preliminary insight into the potential suitability of utilising the LFIS-RA for application in PsA-related foot problems with respect to content validity. Therefore, the objective of this study was to assess the content validity of the LFIS-RA by linking the instrument’s items to the ICF in order to determine the breadth and depth of coverage of concepts important to people with PsA-related foot involvement.

## Methods

**Fig. 1:**
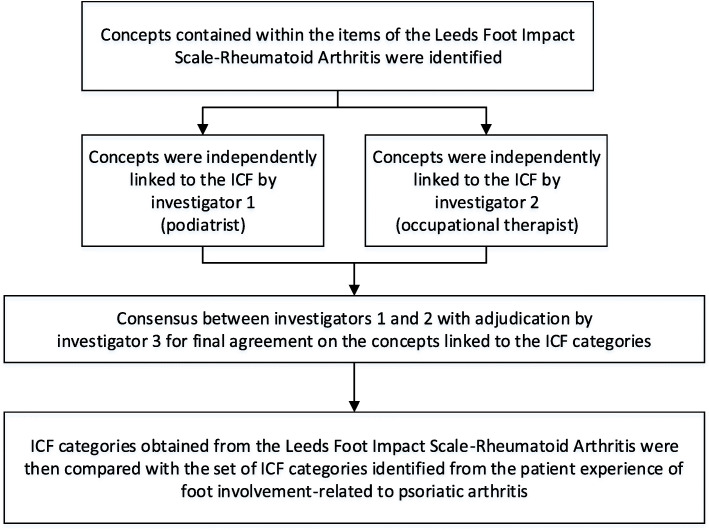
The study design process. *ICF* International Classification of Functioning, Disability and Health

This study was conducted using an iterative consensus-based process of linking items from the LFIS-RA to the ICF classification (Fig. 1) and applies data previously collected by linking concepts, obtained from a qualitative investigation into the patient experience of PsA-foot problems, to the ICF classification [12, 26]. The study was approved by the Ethics committee of each health organisation involved (numbers: HREC/171/LPOOL/353; AUTEC 1/320; RM/3907). Permission was granted from the corresponding author of the LFIS-RA to appraise the content validity in the context of the ICF.

### Linking the LFIS-RA to the ICF

The ICF framework supports the biopsychosocial model of health by recognising the influence of contextual factors on functioning and disability [17]. The ICF classification is divided into four components; Body Structures (s), Body Functions (b), Activities and Participation (d), and Environmental Factors (e) [17]. Within each component, there are multiple categories that are hierarchically grouped within chapters and denoted by unique alphanumeric codes. Within each chapter there are 2nd level, 3rd level and 4th level categories. There is a total of 1454 ICF categories, which are the units of the classification [17].

The LFIS-RA is a self-completed questionnaire comprising 51 items in total, divided into 2 subscales: foot impairment/footwear restriction (LFIS_IF_; items 1 to 21) and activity limitation/participation restriction (LFIS_AP_; items 22 to 51), with dichotomous response options [16]. All concepts within each item of the LFIS-RA were identified and each concept was linked to the best-matched ICF category according to established linking rules [27–29]. Standard linking rules were developed in order to find the most suitable ICF category for each concept and to link to the ICF in a specific and precise manner [27–29]. Using the online ICF classification in its full version, each item was linked to the most precise ICF category [17]. One item could be linked to one or more ICF categories, depending on the number of concepts in the item. The number of categories should be as low as possible but as high as needed to accurately reflect functioning for the particular health condition. For example, the item “my feet get painful when I’m standing” contains the concepts “foot pain” and “standing”, which were linked to the ICF categories “b28015: Pain in lower limb” and “d4154: Maintaining a standing position”.

Concepts recognised as personal factors were linked to the ICF component ‘personal factors’ (pf), because personal factors are not yet specified in ICF categories. If there was insufficient information to make a decision about which ICF category should be linked, it was assigned as not definable (nd). The ‘other specified’ and ‘unspecified’ categories at the end of each chapter were used if a concept was not explicitly specified.

### Agreement analysis

In order to ensure the validity of the linking process, all concepts were independently linked to the ICF by 2 investigators (KC, CT). Both investigators were experienced health professionals with prior knowledge of using the ICF as a classification system [26]. After the independent linking process was complete, consensus between the two investigators was used to determine the final set of categories. In case the investigators could not achieve a consensus, a third investigator was consulted and determined the final category (DET). Investigator professional backgrounds included occupational therapy and podiatry, and all three investigators undertook self-directed training in linking concepts to the ICF supported by the ICF Research Centre [30].

The degree of agreement between the 2 investigators in linking concepts to the ICF was described using the percentage total agreement and the unweighted kappa statistic [31]. Kappa values can range from 0 to 1, where 1 indicates perfect agreement and 0 indicates no additional agreement beyond what is expected by chance alone. When interpreting kappa statistics, published definitions were used with values of less than 0 representing poor agreement, 0.00–0.20 as slight agreement, 0.21–0.40 as fair agreement, 0.41–0.60 as moderate agreement, 0.61–0.80 as substantial agreement, and 0.81–1.00 as almost perfect to perfect agreement [32]. This analysis was performed using SPSS version 25 software (SPSS, Inc., Chicago, IL). Descriptive statistics were used to analyse the number and frequency of ICF categories identified.

### Appraisal of the content validity of the LFIS-RA

Concepts important to people with PsA-related foot involvement were identified in previous research from qualitative data and linked to the full version of the ICF classification [12, 26]. The list of ICF categories serves as a framework that defines the typical spectrum of problems in functioning relevant to the impact of localised disease in the foot in PsA. The ICF categories obtained from the LFIS-RA were systematically compared to the set of ICF categories that were linked to aspects of functioning important and relevant to people with PsA-related foot problems. Frequency and percentage coverage of the ICF categories were calculated in order to determine the extent to which concepts typical in PsA-specific foot involvement are assessed by the LFIS-RA instrument.

## Results

### Linking the LFIS-RA to the ICF

Thirty-five distinct ICF categories from the online classification were linked to the LFIS-RA, which related to Body Structures (*n* = 1, 3%), Body Functions (*n* = 13, 37%), Activities and Participation (*n* = 17, 49%), and Environmental Factors (*n* = 4, 11%) (Table 1). Over half of the ICF categories identified were 2nd level categories (*n* = 18, 51%), followed by 3rd level categories (*n* = 16, 46%) and one 4th level category relating to ‘pain in the lower limb’ (3%). The ICF component that had the most specific categories (higher level) was Activities and Participation, with 13% of concepts being linked to nine 3rd level categories (relating to mobility, undertaking tasks and self-care). This was followed by Body Functions with 8% of concepts being linked to five 3rd level categories (relating to cognitive and muscle function).

**Table 1:** The number and frequency of ICF categories for each component of the ICF classification that were linked to the LFIS-RA and to concepts derived from PsA-related foot involvement

ICF components	ICF categories linked to the LFIS-RA		ICF categories linked to concepts derived from PsA-related foot involvement	
	n (%)	Frequency (%)	n (%)	Frequency (%)
Body Structures	1 (3%)	5 (5%)	17 (12%)	1127 (15%)
Body Functions	13 (37%)	41 (44%)	48 (32%)	2656 (35%)
Activities and Participation	17 (49%)	33 (35%)	55 (37%)	1420 (19%)
Environmental Factors	4 (11%)	15 (16%)	28 (19%)	2327 (31%)
Total	35	94	148	7530

*ICF* international Classification of Functioning, Disability and Health, *LFIS-RA* Leeds Foot Impact Scale in rheumatoid arthritis, *PsA* Psoriatic arthritis

### Agreement analysis

The overall total percentage agreement in the linking of the LFIS-RA to the ICF between the 2 investigators was 77% and the overall kappa statistic was 0.74 (CI 0.67, 0.81). Good levels of interrater agreement were identified across the ICF components in relation to linking to the LFIS_IF_ subscale at 0.79 (CI 0.68, 0.89) and the LFIS_AP_ subscale at 0.67 (CI 0.55, 0.79). In total 22 additional ICF categories were identified between the investigators, which mostly related to cognitive functions (*n* = 6, 27%) and undertaking tasks (*n* = 5, 23%).

### Appraisal of the content validity of the LFIS-RA

Body Structures was the least represented component in the LFIS-RA (3%) with a low frequency of ICF categories relating to the structure of the foot and ankle (*n* = 5, 5%). From the component Body Functions, the most frequent ICF category was b152: Emotional functions (*n* = 11, 12%) and was linked to concepts such as ‘frustrating’, ‘cry’, ‘annoyed’, ‘ashamed’, ‘nervous’, ‘isolated’ and ‘dread’. Pain was covered frequently with descriptors including ‘throb’, ‘hurt’ and ‘burning’ linked to b28015: Pain in the lower limb (*n* = 9, 10%), and discomfort related to ‘pebbles in my shoes’, ‘wakes me up’ and ‘feels heavy’ was linked to other sensory functions. Body Functions representing changes to the spatial-temporal parameters of walking (b770: Gait pattern functions, b455: Exercise tolerance functions) and related dynamic instability (b760: Control of voluntary movement functions) were also frequently cited (Table 2). Activities and Participation was the most represented ICF component in the LFIS-RA (49%) and the most frequent ICF categories were d450: Walking and d230: Carrying out daily routine, which were linked with pain, emotional burden and social withdrawal. The most frequent Environmental Factor was footwear (e1150: *n* = 9, 10%), which was associated with making decisions, walking and self-care activity. In comparison with the most frequent ICF categories linked to PsA-related involvement, the LFIS-RA covered most of the key constructs including pain, functioning, daily activities, footwear restrictions and psychological impact (Table 3).

**Table 2:** The number and frequency of ICF categories for the components Body Structure and Body Function that were linked to the LFIS-RA

ICF categories	LFIS-RA, n (%)
**Body Structure**	–
s7502 Structure of ankle and foot	5 (100%)
**Body Function**	–
b152 Emotional functions	11 (27%)
b28015 Pain in lower limb	9 (22%)
b770 Gait pattern functions	5 (12%)
b760 Control of voluntary movement functions	3 (7%)
b455 Exercise tolerance functions	3 (7%)
b1801 Body image	2 (5%)
b1644 Insight	2 (5%)
b7353 Tone of muscles of lower half of body	1 (2.5%)
b7800 Sensations of muscle stiffness	1 (2.5%)
b134 Sleep functions	1 (2.5%)
b2702 Sensitivity to pressure	1 (2.5%)
b299 Sensory functions and pain, unspecified	1 (2.5%)
b279 Additional sensory functions, other specified and unspecified	1 (2.5%)

*ICF* international Classification of Functioning, Disability and Health, *LFIS-RA* Leeds Foot Impact Scale in rheumatoid arthritis, *PsA* Psoriatic arthritis

**Table 3:** The number and frequency of ICF categories for the components Activities and Participation, and Environmental Factors that were linked to the LFIS-RA

ICF categories	LFIS-RA, n (%)
**Activities and Participation**	–
d450 Walking	7 (22%)
d230 Carrying out daily routine	6 (18%)
d177 Making decisions	3 (9%)
d4551 Climbing	2 (6%)
d4502 Walking on different surfaces	2 (6%)
d2304 Adapting to changes in daily routine	2 (6%)
d4552 Running	1 (3%)
d410 Changing basic body position	1 (3%)
d570 Looking after one’s health	1 (3%)
d4154 Maintaining a standing position	1 (3%)
d920 Recreation and leisure	1 (3%)
d5402 Putting on footwear	1 (3%)
d469 Walking and moving, other specified and unspecified	1 (3%)
d2303 Managing one’s own activity level	1 (3%)
d4602 Moving around outside the home and other buildings	1 (3%)
d799 Interpersonal interactions and relationships, unspecified	1 (3%)
d2202 Undertaking multiple tasks independently	1 (3%)
**Environmental Factors**	–
e1150 General products and technology for personal use in daily living	9 (60%)
e245 Time-related changes	3 (20%)
e115 Products and technology for personal use in daily living	2 (13%)
e399 Support and relationships, unspecified	1 (7%)

*ICF* International Classification of Functioning, Disability and Health, *LFIS-RA* Leeds Foot Impact Scale in rheumatoid arthritis, *PsA* Psoriatic arthritis

Concepts not covered by the LFIS-RA, that were frequently cited by people with PsA-related foot involvement, were the structure and function of skin to the lower limbs and toenails including related self-care activity, and other musculoskeletal structures such as tendons, muscles and fascia (Table 4). Whilst b1801: Body image was covered in the LFIS-RA (*n* = 2, 2%), it was associated with walking changes and negative emotions but not with other relevant disease-specific constructs in PsA including skin and toenail changes, coping strategies and social withdrawal. The LFIS-RA may underestimate the importance of constructs related to self-management strategies in the context of PsA, with limited coverage of coping styles (0%), self-care activity (*n* = 2, 2%) and accessing healthcare (0%). Participation concepts not fully represented in the LFIS-RA included impact on work (d850) and family relationships (d760).

**Table 4:** The most frequent ICF categories linked to concepts derived from PsA-related foot involvement for each ICF component and the extent of coverage by the LFIS-RA

ICF component	ICF category	Concepts derived from PsA-related foot involvement	Percentage coverage by the LFIS-RA, n (%)
Body structures	s75021: Ankle joint and joints foot and toes	‘joints’, ‘ankle joint’, ‘toes’	
	s8104: Skin of lower extremity	‘skin’	
	s8301: Toenails	‘toenails’	
	s7502: Structure of ankle and foot	‘ankle’, ‘heel’, ‘arch’, ‘midfoot’, ‘ball of foot’, ‘forefoot’, ‘sole’	5 (100%)
	s75022: Muscles of ankle and foot	‘tendons’, ‘Achilles tendon’	
	S75023: Ligaments and fascia of ankle and foot	‘plantar fascia’	
Body functions	b28015: Pain in lower limb	‘sore’, ‘throbbing’, ‘sharp’, ‘burning’, ‘aching’, ‘severe’, ‘tender’, ‘unbearable’, ‘unpredictable’, ‘constant’	9 (22%)
	b152: Emotional functions	‘frustrating’, ‘sad’, ‘upset’, ‘embarrassed’, ‘frightening’, ‘envy’, ‘helpless’, ‘depressed’, ‘bad tempered’	11 (27%)
	b1801: Body image	‘revolting’, ‘ugly’, ‘not normal’, ‘I hate the way they look’, ‘disfiguring’, ‘everybody’s eyes goes there’, ‘hide my feet’, ‘I cover up the legs’, ‘don’t want to look outwardly disabled’, ‘it doesn’t feel good’, ‘I don’t want to be noticeable’	2 (5%)
	b810: Protective functions of skin	‘psoriasis’, ‘hard skin’, ‘thin skin’, ‘dry cracked’, ‘splits’, ‘thick skin’, ‘corn’	
	b860: Functions of nails	‘thick’, ‘lift-up’, ‘hard’, ‘pitting’, ‘split’, ‘thin’, ‘discoloured’, ‘wave-shaped’, ‘break off easily’, ‘build-up under the nail’	
	b126: Temperament and personality functions	‘I like to hide my pain’, ‘if I was in pain I would still force myself to participate’, ‘I just put up with it’, ‘plan for my feet and shoes’	
	b770: Gait pattern functions	‘slower’, ‘limping’, ‘shuffle’, ‘hobbling’	5 (12%)
Activities and participation	d450: Walking	‘limited walking activity’, ‘cannot walk barefoot’, ‘painful walking’, ‘cannot walk for long’	7 (22%)
	d5702: Maintaining one’s health	‘you name it I’ve tried it’*,* ‘getting advice and getting feet checked’, ‘I can’t actually look under the sole of my foot’, ‘I try to look after myself as much as I can’	
	d850: Remunerative employment	‘unemployed’, ‘I quit my job, because it was mostly you have to stand’, ‘difficulty sitting for long periods at work’, ‘walking at work’, ‘I want to continue working’	
	d5200: Caring for skin d5204: Caring for toenails	‘moisturising feet’, ‘foot baths’, ‘filing callus’, ‘corns removed’, ‘difficult to cut’, ‘nail polish’, ‘I can’t cut my toenails’	
	d230: Carrying out daily routine	‘can’t do what I want to do’, ‘limits daily activities’, ‘have to keep doing things’, ‘I don’t do much’, ‘difficulty with housework’, ‘makes things difficult’, ‘I can’t do a quarter of the stuff I used to do’, ‘I still have to do what I have to do, I just try and rest in between’	6 (18%)
	d760: Family relationships	‘want to stay healthy for my family and kids’, ‘loss of family time’, ‘burdening the family’, ‘parenting’, ‘affects relationships’, ‘I’m a disappointment to my wife’	
	d920: Recreation and leisure	‘my social life was ruined’, ‘gym’, ‘I used to play soccer’, ‘with my ankle now I can’t exercise’, ‘wedding and formal functions’, ‘stuck at home’, ‘I can’t really go out with friends much’, ‘I don’t like to go out’	1 (3%)
Environmental factors	e1150: General products and technology for personal use in daily living	‘difficulty finding nice looking shoes’, ‘unable to find comfortable shoes’, ‘need wide shoes - bigger size’, ‘cannot wear high-heels’, ‘very limited in the type of shoe’, ‘cannot find suitable shoes for work’, ‘wear the same shoes all the time’, ‘can’t wear open shoes’, ‘closed-in’, ‘need a flexible heel-counter’, ‘can’t wear the clothes you want’	9 (60%)
	e580: Health services, systems and policies	‘accessing podiatry services’, ‘under the care of rheumatology’, ‘delayed diagnosis’	
	e1151: Assistive products and technology for personal use in daily living	‘insoles’, ‘insert’, ‘foot orthotic’	
	e355: Health professionals	‘podiatrist’, ‘rheumatologist’, ‘physiotherapist’, ‘orthopaedic surgeon’	
	e225: Climate	‘summer’, ‘winter’, ‘low-pressure systems’, ‘in summer I find like my foot is quite swollen’	
	e1101: Drugs	‘cortisone injection’, ‘biologics’, ‘pain killers’, ‘steroid creams’	
	e445: Attitudes of strangers	‘other people just don’t realise’, ‘feeling judged’, ‘they think I look healthy’, ‘they don’t understand how it does affect your life with getting up, walking, just simple things’, ‘there’s just no recognition, understanding or acceptance at all’	
	e310: Immediate family	‘my son has got skin psoriasis…a worse state than mine’, ‘thankfully they’re quite understanding’, ‘they helped me a lot’, ‘supportive’	

*ICF* international Classification of Functioning, Disability and Health, *LFIS-RA* Leeds Foot Impact Scale in rheumatoid arthritis, *PsA* Psoriatic arthritis

### Difficulty with linking to the ICF

Three concepts were assigned as not definable referring to quality of life in general; ‘my whole life’ (*n* = 2, 67%) and ‘in the background’ (*n* = 1, 33%). Concepts that were difficult to link to the ICF were related to aspects of time, rest and instability. Although temporal changes associated with pain such as ‘at night’ and ‘end of the day’ were covered in the LFIS-RA and were linked to e245: Time-related changes, other temporal aspects including ‘every time’, ‘all day’ and ‘all the time’ could not be linked to the ICF classification. The concept of rest in the ICF is described as a mental and cardiovascular function, which may not ideally capture the meaning of rest in the context of reduced physical function. Concepts relating to rest in the LFIS-RA such as ‘I have to walk for a bit and sit for a bit’ were linked to b455: Exercise tolerance functions, but reveals deficiencies in the ICF classification. Foot-related instability associated with balance, fear of falling and coordination was linked to b760: Control of voluntary movements. However, instability was difficult to link to a suitable ICF category due to the concept covering various other categories such as stability of joint functions, vestibular and proprioceptive functions, which reflects limitations in the ICF linking process. Coping with PsA-related foot problems was the most frequent concept linked to Personal Factors. Although Personal Factors were not identified in the LFIS-RA, concepts that were covered such as adapting (d2304), planning (d230) and managing activity levels (d2302) could be interpreted as coping strategies if not developed in response to disease impact. This overlap in meaning represents a shortfall of the online ICF classification in defining foot disease burden with possible ambiguity of these concepts reflected as a positive personal attribute or as a negative consequent impairment of the disease.

## Discussion

Using the ICF as a reference, it was possible to assess the content validity of the LFIS-RA in relation to people with PsA-related foot involvement. Although there was coverage of joint-related symptoms, the LFIS-RA may have a limited ability to capture the dermatological impact and site-specific musculoskeletal involvement in PsA. Consequently, hallmark disease features such as skin and nail psoriasis, plantar fasciitis and Achilles enthesitis as well as their impact on footwear characteristics (heel counter, support) may not be well addressed. Whilst there was coverage of constructs relating to pain, emotions, walking, daily activity and footwear, the results of this study indicate that the LFIS-RA may not comprehensively measure the multifaceted impact of foot functional impairments in PsA. Foot involvement can be a major characteristic feature of the disease for a large proportion of people with PsA [33]. A diverse range of disease manifestations can affect the foot in PsA relating to skin, toenails and musculoskeletal structures, which broadens the impact of localised disease on daily activities, social interactions, paid employment and community participation [12].

The LFIS-RA was developed using robust methods in outcome measure design; based on patient-derived statements, an iterative item-reduction process involving a total of 283 patients with rheumatoid arthritis via postal survey, and closely associating components with the ICF framework [16]. The conceptual coverage of domains relevant to PsA-related foot involvement by the LFIS-RA is likely to represent the crossover of concepts typical to foot-specific problems and their impact in both PsA and rheumatoid arthritis [10]. Epidemiological differences between PsA and rheumatoid arthritis may account for some of the concepts that were less well represented by the LFIS-RA. Peak onset of PsA occurs between 30 and 50 years and affects men and women equally [34], compared with the female predominance and older aged onset of rheumatoid arthritis. Rasch analysis was used in the development of the LFIS-RA to remove items influenced by the age, sex and disease duration of respondents, which may account for the reduced coverage of impact domains specific to participation restrictions and body image. For example, a redundant item reported not to fit the Rasch model by gender was ‘I can’t wear my choice of shoe’ [16], but the social stigma of having skin and toenail psoriasis has been shown to limit footwear choice and social participation for both male and female participants with PsA [12]. Furthermore, self-management of foot problems in PsA was considered to influence the severity of, and level of importance attributed to, foot involvement and the consequent impact on daily life [12], but one implication of dichotomised data collected by the LFIS-RA is that these concepts cannot be analysed. Relative importance of foot problems may change over time, in comparison to other structures and in response to adaptation, and these aspects of chronicity related to disease duration may have been removed by the Rasch analysis in the development of the LFIS-RA.

Limited coverage of self-care activity and access to healthcare in the LFIS-RA may reflect the experiences of the study population, who were recruited from sites regarded to be centres of excellence in the UK with an international reputation and where podiatry is embedded into rheumatology services with specialised podiatry roles established. Therefore, the experience of foot care services in the patient cohort from which the LFIS-RA was derived may not be generalisable to Australia or New Zealand (or other countries), where a distinct lack of access to, and provision of, podiatry services and specialist rheumatology services in the public health system have been previously described [35–37].

Scientific research of PsA significantly lags behind that of rheumatoid arthritis [38, 39]. A strong theme from the concepts previously derived from people with PsA-related foot problems [12], but not covered by the LFIS-RA, was the perceived lack of understanding of the disease by others and the consequent importance of supportive relationships and coping strategies such as concealing the disease from others and acquiring knowledge. The ICF categories linked to these concepts related to; the attitudes of others (family, friends, colleagues, strangers and health professionals), temperament and personality functions (b126), family relationships (e310) and Personal Factors [26]. In contrast with rheumatoid arthritis, we are still in a nascent state regarding the evaluation of PsA [40] making illness knowledge and understanding important to people with PsA-related foot involvement.

Patient-reported outcome measures should have evidence of quality criteria, of which content validity is most essential to ensure that an instrument measures all relevant aspects of an outcome [24, 41]. Incorporating the perspective of people with PsA in the development and validation of outcome measures to ensure patients’ concerns are appropriately assessed has been the focus of research led by the Group for Research and Assessment of Psoriasis and Psoriatic Arthritis and the OMERACT [42–46]. The ICF has been used in previous research to determine that concepts derived from patients in qualitative studies are not adequately covered by standardised patient-reported outcome measures used in PsA for assessing functioning [25], which supports the approach taken in deriving the current study results. To better target and treat inflammation present in the foot it is important that the severity, extent and impact of local disease activity is better understood. With no validated patient-reported outcome measures to assess the impact of localised disease in the foot in PsA on functioning and the limitations identified with the content validity of the LFIS-RA, future work should entail the construction of an ICF-based patient-reported outcome measure to assess the impact of PsA-specific foot involvement.

The generalisability of the results from the study is restricted with the original qualitative data generated from participants in Australia and New Zealand. Diverse environmental and personal factors may influence the participation and functioning of people in different countries. To gain cross-cultural insight, further research is needed to explore the international patient perspective and transferability across cultures. In addition, with no established ICF Core Set for foot involvement in PsA the results should be considered as being preliminary. Although the current study adopted a similar approach to other studies investigating the conceptual coverage of outcome measures in PsA [25], the ICF categories of a Core Set should serve as a starting point for such studies having been produced from a programme of work specific to Core Set development [21]. Despite the use of linking rules, individual interpretation of the same item can lead to inconsistencies, as indicated in previous research using the ICF in other rheumatic conditions [47–49]. A consensus-based iterative process and the use of investigators from different professional backgrounds mitigated misinterpretations and improved the quality of the linking process [29]. Both investigators in this study were experienced health professionals with interest in, and knowledge of, the ICF. Limitations of the ICF noted in this study were consistent with previous work [47, 50–54].

## Conclusion

The ICF provides a useful framework for considering the impact of localised disease in the foot on functioning from a holistic biopsychosocial perspective in rheumatic conditions. This study shows that the LFIS-RA has a limited ability to evaluate the full impact of the complex and heterogeneous manifestations of PsA in the foot on aspects of functioning. To incorporate this comprehensive understanding of functioning into the assessment and management of foot problems in PsA, the development of a new foot-specific outcome measure linked to the ICF is required.

## Data Availability

The data that support the findings of this study are available from the corresponding author upon reasonable request.

## Abbreviations

ICF: International Classification of Functioning, Health and Disability
LFIS-RA: Leeds Foot Impact Scale in rheumatoid arthritis
OMERACT: Outcome Measures in Rheumatology
PsA: Psoriatic arthritis
